# Method of levels therapy for first-episode psychosis: rationale, design and baseline data for the feasibility randomised controlled Next Level study

**DOI:** 10.1192/bjo.2018.44

**Published:** 2018-08-14

**Authors:** Robert Griffiths, Warren Mansell, Timothy A. Carey, Dawn Edge, Richard Emsley, Sara J. Tai

**Affiliations:** NIHR Clinical Doctoral Research Fellow and Psychological Therapist, School of Psychological Sciences, The University of Manchester and Psychosis Research Unit, Greater Manchester Mental Health NHS Foundation Trust, UK; Reader in Clinical Psychology, School of Psychological Sciences, The University of Manchester, UK; Director, Centre for Remote Health, Flinders University, Australia; Senior Lecturer, School of Psychological Sciences, The University of Manchester, UK; Professor of Medical Statistics & Trials Methodology, Biostatistics and Health Informatics Department, Institute of Psychiatry, King's College London, UK; Senior Lecturer in Clinical Psychology, School of Psychological Sciences, The University of Manchester, UK

**Keywords:** Method of Levels, first-episode psychosis, randomised controlled trial, feasibility

## Abstract

**Background:**

Method of levels (MOL) is an innovative transdiagnostic cognitive therapy with potential advantages over existing psychological treatments for psychosis.

**Aims:**

The Next Level study is a feasibility randomised controlled trial (RCT) of MOL for people experiencing first-episode psychosis. It aims to determine the suitability of MOL for further testing in a definitive trial (trial registration ISRCTN13359355).

**Method:**

The study uses a parallel group non-masked feasibilityRCT design with two conditions: (a) treatment as usual (TAU) and (b) TAU plus MOL. Participants (*n* = 36) were recruited from early intervention in psychosis services. Outcome measures are completed at baseline, 10 and 14 months. The primary outcomes are recruitment and retention.

**Results:**

Participants’ demographic and clinical characteristics are presented along with baseline data.

**Conclusions:**

Next Level has recruited to target, providing evidence that it is feasible to recruit to a RCT of MOL for first-episode psychosis.

**Declaration of interest:**

None.

## Background

People experiencing a first episode of psychosis (FEP) should have access to early intervention in psychosis (EIP) services that are capable of delivering appropriate psychological interventions, including cognitive–behavioural therapy for psychosis (CBTp).^1^^,^^2^ There is relatively good evidence to support the use of CBTp, with most meta-analyses estimating effect sizes in the small to moderate range.^3^^–^^5^ However, despite recommendations from the National Institute for Health and Care Excellence that everyone experiencing psychosis should be offered CBTp, levels of implementation have remained low.^6^^–^^8^ A recent audit of EIP services found that just 41% of patients had been offered CBTp and, of these, only 51% accepted the offer.^9^ Additionally, evidence from qualitative studies suggests that some patients find CBTp ‘difficult to engage with’ and ‘emotionally difficult’.^10^^,^^11^ There is also evidence that CBTp has only modest benefits over other ‘generic’ interventions for people experiencing psychosis, such as befriending and supportive counselling.^4^

There are high levels of comorbidity among people who experience psychosis, including problems with anxiety, depression, post-traumatic stress disorder and substance misuse.^12^^–^^16^ This presents challenges for clinicians aiming to deliver disorder-specific interventions for psychosis. Where individuals prioritise non-psychotic difficulties, CBTp practitioners will draw on relevant, compatible approaches to inform treatment.^17^ However, clinicians require suitable training and supervision in the application of these approaches. There is also good evidence that transdiagnostic cognitive and behavioural processes maintain psychological distress across diagnostic categories.^18^^,^^19^ Interventions that specifically target transdiagnostic processes might have advantages over disorder-specific approaches in terms of efficiency^20^ and the extent to which they meet the complex needs of people who experience psychosis.^21^^,^^22^

Some researchers have advocated focusing on single symptoms of psychosis (such as persecutory delusions) and targeting the mechanisms proposed to be maintaining them (for example worry, reasoning biases).^23^^,^^24^ Here we present an alternative approach to psychotherapy that targets goal conflict, a core mechanism proposed to maintain psychological distress across diagnostic categories, irrespective of the exact symptoms or problems reported by individuals.

## Method of levels

The method of levels (MOL) is a transdiagnostic cognitive therapy that has been well described in a number of treatment manuals.^25^^–^^27^ MOL aims to help people shift their awareness onto the conflicted goals that are believed to be maintaining distress. Sustaining awareness on the conflict facilitates an innate learning process called reorganisation, which enables people to resolve goal conflict. Therapists delivering MOL have two goals: (a) to encourage the person to talk freely about their problems, and (b) to pay attention for ‘disruptions’ and, when they occur, ask about these. Disruptions are indications that the person's awareness has fleetingly shifted onto potentially relevant background thoughts. Examples would include interruptions to the flow of speech, smiling or laughing, and evaluative statements (such as ‘That sounds silly’).

MOL has several potential advantages over existing psychological approaches for psychosis. These include: it is applicable to individuals presenting with multiple problems simultaneously; it aims to give people greater choice and control over the interventions they receive; treatment is tailored to meet their individual needs; it is based on clear principles derived from a robust scientific theory of human behaviour (see below for more details); it has the potential to use psychological resources more efficiently; and it aims to directly target the factor that is proposed to maintain psychological distress.

## Study aims

To summarise, although CBTp is the current ‘gold standard’ psychological intervention for individuals using EIP services, there are significant difficulties with its implementation in routine clinical practice, as well as queries about how effectively and efficiently it is meeting the psychological needs of this population. MOL is an innovative and promising psychological therapy for FEP. It has several potential advantages over existing treatments and warrants further evaluation.^21^^,^^22^ No previous trials of MOL for FEP have been conducted, so it is necessary to establish the feasibility of recruitment and retention before a full efficacy trial could be justified.

This study aims to answer four research questions.

- Is it feasible to recruit and retain people experiencing a first episode of psychosis in a randomised controlled trial (RCT) of MOL?
- Is MOL an acceptable psychological intervention for people experiencing FEP?
- Is it feasible to deliver MOL to people experiencing FEP?
- Are adaptations necessary to overcome problems or barriers to the implementation of MOL in EIP services?

## Method

Next Level is a parallel group feasibility RCT with two conditions: (a) treatment as usual (TAU) and (b) TAU plus MOL. The study was prospectively registered with the ISRCTN registry (trial registration ISRCTN13359355). As a feasibility trial, participants and their clinical team, the trial therapist and the outcome assessor are not masked to group allocation. If MOL appears suitable for further testing in an efficacy trial, outcome assessors would be masked to group allocation. A trial retention rate of 80% at final follow-up would be considered a successful outcome. The trial was designed with reference to the Medical Research Council's guidelines on developing and evaluating complex interventions.^28^ The trial is sponsored by the University of Manchester and is being conducted across two EIP services within Greater Manchester Mental Health NHS Foundation Trust. Participants randomised to TAU continue to receive support from their EIP team. Participants randomised to the treatment group receive MOL in addition to their usual care. A novel feature of this design is that participants in the treatment group are able to choose the number, frequency and duration of MOL sessions they access over the course of the treatment window. This approach is consistent with the underlying theoretical assumptions of MOL. A nested qualitative study is also included in the design.

### Trial oversight and ethical approval

A trial steering committee (TSC) comprising clinical, academic and patient members has been convened to oversee the study. As a small feasibility study, it was not deemed necessary to establish a separate data monitoring and ethics committee. Instead, the TSC also served some of the functions normally carried out by a data monitoring and ethics committee. The independent members of the TSC had the option to meet independently of the research team if required.

Ethical approval was received from the North West – Greater Manchester Central Research Ethics Committee prior to commencing recruitment (REC reference: 16/NW/0592; IRAS project ID: 204043).

### Randomisation

Following baseline assessments, participants were randomised by R.G. to one of the two conditions in a ratio of 1:1. An online randomisation service (Sealed Envelope Ltd, 2017, https://www.sealedenvelope.com/simple-randomiser/v1/) was used to allocate participants in random permuted blocks. There was no stratification of the study sample. To minimise the potential for bias, participants were randomised in the order they completed baseline assessments.

### Sample size

A formal power calculation was not performed, since the aim of the study is not to estimate between-group treatment effects. A sample size of 15 participants or more in each group is considered adequate for a feasibility RCT.^29^ To allow for potential attrition, the recruitment target was 36 participants.

### Recruitment

Participants were recruited from two EIP services based in a single National Health Service (NHS) trust in the UK. Participants were either experiencing or recovering from a recent FEP. Care coordinators within those teams were asked to raise awareness of the study among patients on their case-loads. Presentations, posters and leaflets were used to provide study information to care coordinators. Individuals who expressed an interest in the study were contacted via telephone by the chief investigator. If verbal consent was given at this point, patients were invited to meet the chief investigator in person to receive information about the study, complete a brief eligibility screen and provide written consent to participate in the study prior to the completion of baseline assessments. Participants were made aware of their right to withdraw from the study at any time.

### Inclusion criteria

- People aged 16–65 years.
- Current user of Greater Manchester Mental Health NHS Foundation Trust early intervention services.
- Sufficient English language abilities (verbal and written) to complete written material (for example, outcome measures) and participate in psychological therapy.
- Willing and able to provide informed consent.

### Exclusion criteria

- People aged under 16 or over 65 years of age.
- Not currently using Greater Manchester Mental Health NHS Foundation Trust early intervention services.
- Literacy or English language difficulties that make it difficult for the person to complete written material (for example, outcome measures) or to participate in psychological therapy.
- Individuals currently serving custodial prison sentences.

### Eligibility and outcome measures

A brief screen consisting of the inclusion and exclusion criteria was used to ensure eligibility. A summary of the assessment schedule is presented in the Appendix. Assessments in both arms were completed by the chief investigator, who was not masked to group allocation.

The proposed primary clinical outcome measure for the purposes of estimating an effect size is the Psychological Outcome Profiles (PSYCHLOPS),^30^ a participant-generated outcome measure that assesses well-being, functioning and distress. Cronbach's alpha in a clinical sample was 0.81,^31^ demonstrating satisfactory internal reliability.

The CORE Outcome Measure (CORE-OM)^32^ is a 34-item self-report instrument that assesses the four domains of subjective well-being, symptoms, functioning and risk. It shows good sensitivity to change and has been used in a variety of practice settings. Cronbach's alpha in clinical samples was found to be 0.94, indicating satisfactory internal reliability.^32^

The Reorganisation of Conflict Scale (ROC)^33^ is a 22-item self-report measure. Each item is scored on a scale of 0 (‘I don't believe this at all’) to 100 (‘I believe this completely’). The study used an 11-item subscale of the ROC that has previously shown satisfactory internal reliability, with a Cronbach's alpha of 0.83.^34^ The subscale measures the components of goal conflict reorganisation, which is proposed to be the key mechanism of change in MOL.

The Questionnaire about the Process of Recovery (QPR)^35^ is a 22-item self-report questionnaire developed in collaboration with patients. It is designed to measure personal recovery from psychosis on two subscales: intrapersonal functioning and interpersonal functioning. Cronbach's alpha was found to be 0.94 for the intrapersonal scale and 0.77 for the interpersonal scale, indicating good internal consistency.^35^

The Outcome Rating Scale (ORS)^36^ is a visual analogue questionnaire that assesses functioning in four domains: individual, social, relational and overall functioning. It is scored from 0 to 40, with scores at or below 25 indicating clinically severe levels of psychological distress. Cronbach's alpha was found to be 0.93, indicating good internal consistency.^36^

The Session Rating Scale (SRS)^37^ is also a visual analogue scale. It assesses patients' perceptions of the therapeutic alliance, including the extent to which the participant felt respected and heard. As with the ORS, the SRS is scored from 0 to 40 with scores of 36 or below indicating cause for concern about the therapeutic alliance. Cronbach's alpha was found to be 0.88, indicating satisfactory internal consistency.^37^

### MOL intervention

MOL directly applies the principles of a robust theory of human behaviour called perceptual control theory (PCT) to the practice of psychotherapy. Detailed descriptions of PCT and its application to the delivery of psychological therapy are available.^38^^,^^39^ The proposed mechanism of change in MOL is the reorganisation of goal conflict. MOL therapists aim to guide a person's awareness onto the source of the conflict, facilitating this process. MOL is a transdiagnostic psychological intervention, which means it is applicable to individuals presenting with diverse problems, irrespective of any diagnosis they might have received. Sessions typically last between 15 and 60 min.

Because PCT proposes that change is non-linear and idiosyncratic,^27^ participants are expected to require a different number and frequency of sessions over the course of the 10-month treatment window. To support this, the study uses patient-led appointment scheduling, an approach that has already been used successfully with a secondary care population.^20^ MOL sessions will be offered at two community venues, one at each recruitment site. A total of five appointment slots will be made available each week at the site with higher levels of recruitment. The site with lower recruitment will have three appointment slots available each week. Capacity issues will be reviewed on an ongoing basis to monitor whether additional appointment slots are required. Participants allocated to receive MOL will be able to book the sessions they require using SMS (short message service) messages, telephone calls or a dedicated online appointment booking website. There is no minimum or maximum number of sessions that participants are expected to attend over the 10-month treatment window where sessions are available to them. Detailed descriptions of MOL and patient-led scheduling are available in several treatment manuals.^25^^–^^27^^,^^40^

The MOL sessions are delivered by the study's chief investigator, a suitably trained clinician with experience of delivering psychological interventions for psychosis. The clinician receives weekly supervision from experienced MOL practitioners. The MOL intervention is delivered according to the guidelines described in published treatment manuals.^25^^–^^27^ Audio recordings of MOL sessions are rated using the MOL Session Evaluation Form.^41^ To ensure fidelity to the approach, a randomly selected sample of MOL session audio recordings will be independently rated at the end of the trial. The intention is to rate 20% of audio recordings.

### Safety monitoring and reporting

Untoward occurrences that result in death, admission to hospital, disability, that are considered life threatening or are otherwise deemed medically significant will be recorded. Incidences of threatened or actual overdose, self-harm or harm to others will also be recorded. Potential adverse events are most likely to be identified at MOL sessions, assessments and qualitative interviews. In addition, participants' medical notes will be reviewed at the end of the trial to identify any other potential adverse events. In the first instance, the chief investigator, primary investigator and chair of the TSC will review potential adverse events to determine the appropriate response. All potential adverse events will be reviewed by the TSC.

### Statistical analysis

Statistical analysis began once all follow-up data had been collected in June 2018. Reporting will adhere to the CONSORT guidelines for pilot and feasibility studies^42^ and will include study attrition and follow-up rates.

### Primary outcomes

Analysis will involve tabulated and graphical summaries of the primary feasibility and acceptability outcome measures. Summary statistics will include the number of individuals who expressed an interest in the trial, the proportion of potentially eligible participants who consented to take part, trial drop-out and the number of participants retained at 10- and 14-month follow-up.

In line with the findings of a recent meta-analysis of attrition rates in complex interventions for schizophrenia,^43^ a retention rate of 80% within the study as a whole would be considered a successful outcome, 70% would be considered borderline and below 60% would be considered an unacceptably low retention rate.

The study's use of patient-led scheduling means it will not be possible to determine drop-out from the MOL intervention prior to the 10-month follow-up assessment. Summary statistics will be presented showing the average number of MOL sessions attended by participants, the number of participants who attended no MOL sessions, and the number of cancelled and missed appointments. Details of any other psychological interventions received by participants in both arms of the trial will also be reported. Data from the nested qualitative study will also contribute to answering the questions regarding the feasibility and acceptability of the trial design and MOL intervention.

### Secondary outcomes

To inform potential effect sizes for a future definitive trial, linear regression will be used to examine the effect of treatment group allocation on outcome measures at post-treatment, adjusting for outcome measures at baseline. The PSYCHLOPS will be treated as the primary clinical outcome measure for this purpose. However, because effect sizes calculated from feasibility trials with fewer than 35 participants in each arm are likely to be unreliable,^44^ results will be treated with caution and 95% confidence intervals for effect sizes will be considered to check if a minimal clinically important difference is within the interval. Point estimates and associated 95% confidence-intervals of effect sizes will be reported rather than statistical significance (*P*-values). Every effort will be made to follow-up participants in both arms for assessments, and the analysis will use, where appropriate, statistical techniques for handling missing data. Statistical analysis will be conducted in accordance with the principles of intention-to-treat analysis. Data from participants in the treatment group who attend varying numbers of MOL sessions (including those who attend no sessions) will be included in the final statistical analysis.

## Results

**Fig. 1:**
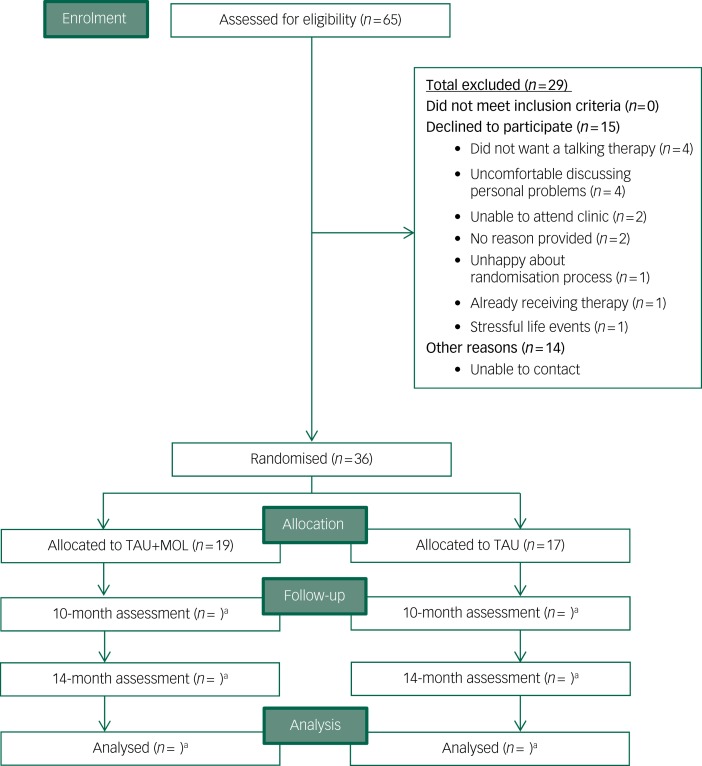
CONSORT diagram. TAU, treatment as usual; MOL, method of levels. a. *n* for follow-up and analysis not yet available.

Recruitment to the Next Level trial began in September 2016 and ended when the target of *n* = 36 was met in April 2017. The randomisation procedure allocated 19 participants to TAU + MOL and 17 to TAU. Participants were recruited at a rate of 4.5 a month from two EIP services, with combined team case-loads of 283 patients. A total of 65 patients (approximately 23% of all potentially eligible individuals across the two EIP services) expressed an interest in participating in the study to their care coordinator. Relatively few individuals declined to participate after expressing an interest in the study (*n* = 15). The most common reasons for declining were not wanting a talking therapy (*n* = 4) or feeling uncomfortable about discussing personal details (*n* = 4). Just one potential participant declined because of concerns about the randomisation process. It was not possible to contact a proportion (*n* = 14) of those individuals who were potentially eligible and had expressed an interest in the study to their care coordinators. A CONSORT^42^ diagram is presented in Fig. 1.

Recruitment was significantly higher from one of the two EIP services, with *n* = 31 (86.1%) of trial participants using that service. The number of potential participants identified by individual care coordinators ranged from *n* = 0 to *n* = 15. It is not clear what overall proportion of eligible patients were made aware that they could participate in the study.

A summary of participant characteristics is presented in Table 1. Participants' clinical characteristics are shown in Table 2. The study has a relatively young and predominantly male sample. The majority of participants are White British, single, living in mainstream accommodation and unemployed. Participants generally had a relatively long duration of untreated psychosis (DUP) prior to being accepted to the EIP service supporting them. Average DUP was noticeably longer in the TAU + MOL arm of the study. On average, participants had been accepted by the EIP service just over 13 months prior to being accepted into the study. The most frequently occurring primary diagnosis across both groups was one of psychosis spectrum disorder. However, many participants had received other primary diagnoses, including common mental health problems, such as anxiety and depression. A proportion had received no diagnosis at all. Most participants were prescribed antipsychotic medication and just over half were prescribed antidepressants. A minority of participants were not prescribed any psychotropic medication. A summary of baseline statistics is presented in Table 3.

**Table 1:** Participant characteristics by randomisation group

		TAU + MOL (*n* = 19)	TAU (*n* = 17)	Total (*N* = 36)
Age (years), mean (S.D.)		32.2 (13.1)	28.9 (7.3)	30.6 (10.7)
Gender	Male	11 (57.9%)	12 (70.6%)	23 (63.9%)
	Female	8 (42.1%)	5 (29.4%)	13 (36.1%)
Ethnicity	White – British	18 (94.7%)	14 (82.4%)	32 (88.9%)
	White – any other white background	0 (0.0%)	1 (5.9%)	1 (2.8%)
	Mixed – any other mixed background	1 (5.3%)	0 (0.0%)	1 (2.8%)
	Asian/Asian British	0 (0.0%)	1 (5.9%)	1 (2.8%)
	Black/Black British – African	0 (0.0%)	1 (5.9%)	1 (2.8%)
Civil status	Single	14 (73.7%)	10 (58.8%)	24 (66.7%)
	Married/cohabiting	5 (26.3%)	5 (29.4%)	10 (27.8%)
	Divorced/separated	0 (0.0%)	2 (11.8%)	2 (5.6%)
Accommodation	Mainstream housing	18 (94.7%)	15 (88.2%)	33 (91.7%)
	Homeless	1 (5.3%)	1 (5.9%)	2 (5.6%)
	Supported housing	0 (0.0%)	1 (5.9%)	1 (2.8%)
Employment status	Unemployed	9 (47.4%)	10 (58.8%)	19 (52.8%)
	Paid employment	5 (26.3%)	3 (17.6%)	8 (22.2%)
	Education/training	3 (15.8%)	4 (23.5%)	7 (19.4%)
	Unpaid employment	2 (10.5%)	0 (0.0%)	2 (5.6%)

**Table 2:** Clinical characteristics of participants

	TAU + MOL (*n* = 19)	TAU (*n* = 17)	Total (*n* = 36)
Duration of untreated psychosis, months: mean (s.d.)	46.1 (60.8)	14.6 (21.8)	30.9 (48.2)
Length of EIP treatment, months: mean (s.d.)	10.5 (9.5)	16.2 (11.7)	13.2 (10.8)
Primary diagnosis, *n* (%)			
Psychosis spectrum disorders	5 (26.3)	6 (35.3)	11 (30.6)
Depression	2 (10.5)	4 (23.5)	6 (16.7)
Bipolar disorders	1 (5.3)	1 (5.9)	2 (5.6)
Anxiety disorders	2 (10.5)	1 (5.9)	3 (8.3)
Mixed anxiety and depression	2 (10.5)	1 (5.9)	3 (8.3)
Substance misuse disorders	2 (10.5)	1 (5.9)	3 (8.3)
Eating disorder	0 (0)	1 (5.9)	1 (2.8)
Personality disorders	0 (0)	1 (5.9)	1 (2.8)
No diagnosis	5 (26.3)	1 (5.9)	6 (16.7)
Medication, *n* (%)			
Antipsychotics	11 (57.9)	13 (76.5)	24 (66.7)
Antidepressants	9 (47.4)	10 (58.8)	19 (52.8)
Other psychotropic	4 (21.1)	4 (23.5)	8 (22.2)
No psychotropic	7 (36.8)	3 (17.6)	10 (27.8)

TAU, treatment as usual; MOL, method of levels; EIP, early intervention in psychosis.

**Table 3:** Summary of baseline statistics

	Mean (s.d.)	
	TAU + MOL (*n* = 19)	TAU (*n* = 17)
Primary outcome		
Psychological Outcome Profiles, total	16.3 (3.5)	14.9 (2.6)
Secondary outcomes		
CORE Outcome Measure, total	71.1 (25.7)	60.3 (24.2)
Outcome Rating Scale, total	14.0 (10.0)	19.1 (9.2)
Questionnaire about the Process of Recovery, total	45.7 (12.9)	54.6 (13.0)
Reorganisation of Conflict Scale, total	58.9 (20.1)	69.1 (14.6)

TAU, treatment as usual; MOL, method of levels.

## Discussion

This is the first RCT of MOL for people experiencing FEP. The results will be used to answer questions about the feasibility and acceptability of delivering MOL to people experiencing FEP. The issue of whether adaptations are required to deliver the intervention to people using EIP services will also be considered. The answers to these questions will inform decision-making regarding the suitability of MOL for further testing in a definitive trial.

### Main findings and interpretation

The relatively long DUP of the sample, particularly in the TAU + MOL arm, indicates that participants had experienced symptoms of psychosis for a significant amount of time prior to acceptance by an EIP team. Longer DUP is associated with greater overall symptoms, lower functioning and poorer quality of life.^45^ The study sample's lack of ethnic diversity potentially limits the generalisability of results. The sample included participants with a diverse range of diagnoses, including some who had not received any diagnosis. This is likely to be a reflection of the fact that EIP services are commissioned to work with individuals where there is ‘diagnostic uncertainty’.^46^

These initial data suggest that it is possible to recruit and randomise participants to an RCT of MOL for FEP. Recruitment to target took just over 7 months at an average rate of 4.5 participants per month, which was considered a successful outcome. At least three factors are likely to account for the disparity in recruitment rates between the two EIP services. First, the study was open to referrals 2 months earlier at the service with higher recruitment. Second, the site with lower recruitment is commissioned to work with significantly fewer patients (98 compared with 185). Third, the chief investigator previously worked as a clinician in the EIP service with higher recruitment, so this is also likely to be a factor. This is consistent with research suggesting that clinicians are more likely to refer patients to clinical trials where they trust the investigators conducting the study.^47^

Future trials should consider methods of establishing trust between clinicians and researchers in order to facilitate appropriate referrals. Another issue to consider in future trials is ensuring that all potentially eligible participants are made aware of relevant research. For ethical reasons, this study relied on care coordinators to identify potentially eligible participants. However, there was a high degree of variation in the referral rates between individual care coordinators. This suggests that care coordinators acted as ‘gatekeepers’ to the trial, potentially limiting patient choice about trial participation. Perhaps a more ethical approach in future trials would be to approach potential participants directly, with the aim of increasing equitable access.

The inclusion and exclusion criteria for this study closely matched those used by the EIP services where recruitment took place. This explains why no potential participants were deemed ineligible for the study. Relatively few participants declined to take part in the study, providing prima facie evidence that the majority of participants find the idea of randomisation acceptable.

Because the PSYCHLOPS measures within-person change related to an idiosyncratic participant-defined problem, it is not possible to interpret the baseline data in isolation. Change can only be measured meaningfully when compared with the post-therapy scores that will be collected during follow-up. This also applies to the QPR and ROC measures. The mean CORE-OM scores for the TAU + MOL and TAU arms indicate ‘moderate to severe’ and ‘moderate’ levels of distress, respectively. The mean ORS scores were below 25 for both study arms, indicating clinically severe levels of psychological distress.

### Limitations

One potential limitation of this study is the lack of specific measures of psychotic symptoms. The majority of recent RCTs for psychosis include a measure of psychotic symptoms as their primary outcome.^48^ The rationale for not including a measure of psychotic symptoms is that MOL aims to reduce underlying psychological distress, rather than reduce or remove the symptoms of psychosis. Outcome measures that are consistent with the stated aims of MOL were selected. Including psychosis-specific measures, although potentially yielding some useful data, would have added to participant burden. Additionally, the approach taken here is consistent with the argument that it is more appropriate for psychological interventions for psychosis to focus on ameliorating psychological distress rather than symptom reduction.^49^^,^^50^

Participants in both groups might be offered psychological interventions as part of the routine care they receive from their usual EIP service. However, participants in the TAU group were not systematically offered access to any psychological interventions as part of their involvement with this study. If there is differential access to psychological interventions between groups it could be considered a limitation of this study.

Data collection was completed as planned in June 2018. Once analysed, results will be published in peer reviewed journals and presented at relevant conferences. Results will be presented in line with the primary and secondary outcomes specified in this paper.

## Appendix

### Assessment schedule summary

**Table:**

	Baseline	MOL session measures	10 months	14 months
Psychological Outcome Profiles	✓		✓	✓
CORE Outcome Measure	✓		✓	✓
Reorganisation of Conflict Scale	✓	✓	✓	✓
Questionnaire about the Process of Recovery	✓		✓	✓
Outcome Rating Scale	✓	✓	✓	✓
Session Rating Scale		✓		
